# A FAM83A Positive Feed-back Loop Drives Survival and Tumorigenicity of Pancreatic Ductal Adenocarcinomas

**DOI:** 10.1038/s41598-019-49475-5

**Published:** 2019-09-16

**Authors:** Neetha Parameswaran, Courtney A. Bartel, Wilnelly Hernandez-Sanchez, Kristy L. Miskimen, Jacob M. Smigiel, Ahmad M. Khalil, Mark W. Jackson

**Affiliations:** 1 Department of Pathology, Case Western Reserve University, 2103 Wolstein Research Building, Cleveland, OH 44106 USA; 2 Department of Pharmacology, Case Western Reserve University, 2103 Wolstein Research Building, Cleveland, OH 44106 USA; 3 Department of Epidemiology and Biostatistics, Case Western Reserve University, 2103 Wolstein Research Building, Cleveland, OH 44106 USA; 4 Department of Genetics and Genome Sciences, Case Western Reserve University, 2103 Wolstein Research Building, Cleveland, OH 44106 USA; 5 Case Comprehensive Cancer Center, Case Western Reserve University, 2103 Wolstein Research Building, Cleveland, OH 44106 USA

**Keywords:** Pancreatic cancer, Oncogenes, Growth factor signalling, Mechanisms of disease

## Abstract

Pancreatic ductal adenocarcinomas (PDAC) are deadly on account of the delay in diagnosis and dearth of effective treatment options for advanced disease. The insurmountable hurdle of targeting oncogene KRAS, the most prevalent genetic mutation in PDAC, has delayed the availability of targeted therapy for PDAC patients. An alternate approach is to target other tumour-exclusive effector proteins important in RAS signalling. The Family with Sequence Similarity 83 (FAM83) proteins are oncogenic, tumour-exclusive and function similarly to RAS, by driving the activation of PI3K and MAPK signalling. In this study we show that *FAM83A* expression is significantly elevated in human and murine pancreatic cancers and is essential for the growth and tumorigenesis of pancreatic cancer cells. Elevated FAM83A expression maintains essential MEK/ERK survival signalling, preventing cell death in pancreatic cancer cells. Moreover, we identified a positive feed-forward loop mediated by the MEK/ERK-activated AP-1 transcription factors, JUNB and FOSB, which is responsible for the elevated expression of oncogenic *FAM83A*. Our data indicates that targeting the MEK/ERK-FAM83A feed-forward loop opens up additional avenues for clinical therapy that bypass targeting of oncogenic KRAS in aggressive pancreatic cancers.

## Introduction

Pancreatic cancer has the highest mortality of all cancers, with less than 9% of the patients surviving beyond five years^1^. The majority of patients present with advanced disease that has already metastasized, and typically, pancreatic cancer cells are remarkably resistant to chemotherapy or rapidly acquire resistance following treatment^2–4^. Of concern, population forecasts from the United Nations (UN) predict a continued rise in pancreatic cancer incidences worldwide in the coming decades^5^. Current treatment strategies include a combination of surgery to remove localized tumours, single agent Gemcitabine therapy and radiation. The only targeted therapy approved for pancreatic cancers is the epidermal growth factor receptor (EGFR) inhibitor Erlotinib, which is administered in combination with Gemcitabine and results in a modest increase in overall survival^6,7^. Once a patient progresses on therapy, options remain limited. Clearly, new treatments are urgently needed to extend the lives of patients with pancreatic cancer.

More than 95% of pancreatic ductal adenocarcinomas (PDAC) are driven by mutation in the Kirsten rat sarcoma (*KRAS)* gene^8,9^, which constitutively activates rapidly accelerated fibrosarcoma (RAF)/mitogen activated protein kinase kinase (MEK)/extracellular signal-regulated kinases (ERK) and phosphoinositide 3-kinase (PI3K)/AKT/mammalian target of rapamycin (mTOR) signalling. For 30 years, strategies aimed at directly targeting mutant RAS have proven unsuccessful, earning RAS the reputation of being undruggable^10^. However, recent research identifying small molecules that inhibit protein interactions of RAS bring hope to this long-drawn struggle^11^. For example, DeltaRasin is a small molecule inhibitor that blocks the interaction of RAS with phosphodiesterase- delta subunit (PDEδ), altering RAS localization and inhibiting its function^12^. The RAS-mimetic Rigosertib binds to RAS-binding domain (RBD) of RAF blocking its functional interaction with RAS and inhibiting downstream MEK/ERK effector signaling^13^. Moreover, improved analogues of mutant KRAS-G12C-specific inhibitors such as ARS853 are currently being optimized to directly target RAS^14^. Nonetheless, the clinical efficacy of the newer drugs remains uncertain and it is essential that newer ‘druggable’ targets are identified that can bypass the need to target/inhibit oncogenic RAS.

We discovered the Family with Sequence Similarity 83 (FAM83) proteins in a forward genetic screen for genes that promote cellular transformation. In mammary epithelial cells FAM83 proteins function similarly to RAS, driving the activation of PI3K and MAPK signaling^15,16^. The FAM83 protein family has 8 members varying greatly in size, with each sharing a conserved amino-terminal Domain of Unknown Function (DUF1669 domain). FAM83 proteins are elevated in many human cancers and have been implicated in cancer growth, metastasis and therapy resistance^17,18^. The smallest FAM83 member, FAM83A, was separately identified by the Bissell laboratory, due to its ability to promote EGFR Tyrosine Kinase Inhibitor (TKI) resistance, also in mammary epithelial cells^19^. FAM83A is overexpressed in many human cancers and yet it remains unclear what factors drive elevated FAM83A expression in the transformed cells.

In the current study, we show that *FAM83A* expression is significantly elevated in human and murine pancreatic cancers. Ablation of FAM83A expression suppresses MEK/ERK survival signalling, resulting in apoptosis and suppression of tumorigenicity. Importantly, FAM83A expression is regulated by the activating protein-1 (AP-1) transcription factors, Jun proto-oncogene B (JUNB) and FBJ murine osteosarcoma viral oncogene homolog B (FOSB), which are activated by MEK/ERK activity. We propose that the MEK/ERK-mediated induction of FAM83A gene expression creates a positive, feed-forward loop involving FAM83A and RAS effectors ultimately drive pancreatic cancer cell survival. Our studies provide new insight into RAS-driven pancreatic cancer development and progression and serve as the foundation for developing new therapies capable of targeting RAS/FAM83A signalling axis.

## Results

### FAM83A expression is elevated in pancreatic cancers

**Figure 1:**
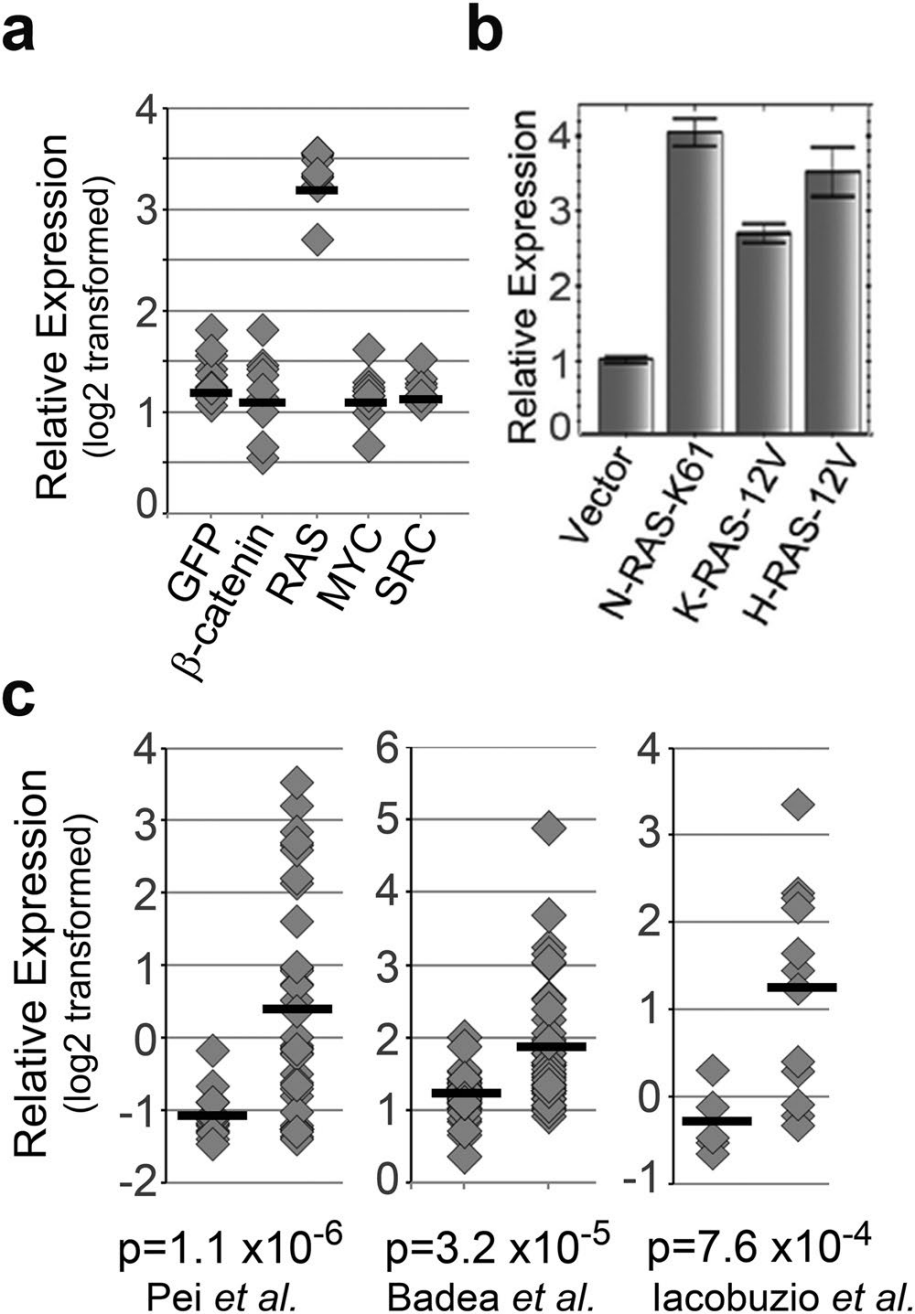
FAM83A expression is elevated in pancreatic cancers. (**a**) Microarray data showing *FAM83A* gene expression in human mammary epithelial cells expressing known oncogenes^20^. (**b**) Constitutively-active mutants of *NRAS*, *KRAS* or *HRAS* were expressed in human mammary epithelial cells and *FAM83A* mRNA expression was assessed by real-time PCR. (**c**) Oncomine microarray data showing *FAM83A* mRNA expression in pancreatic cancer tissue compared to normal human pancreas^21–23^. Student’s t-test was used to assess statistical significance and p-values.

**Figure 2:**
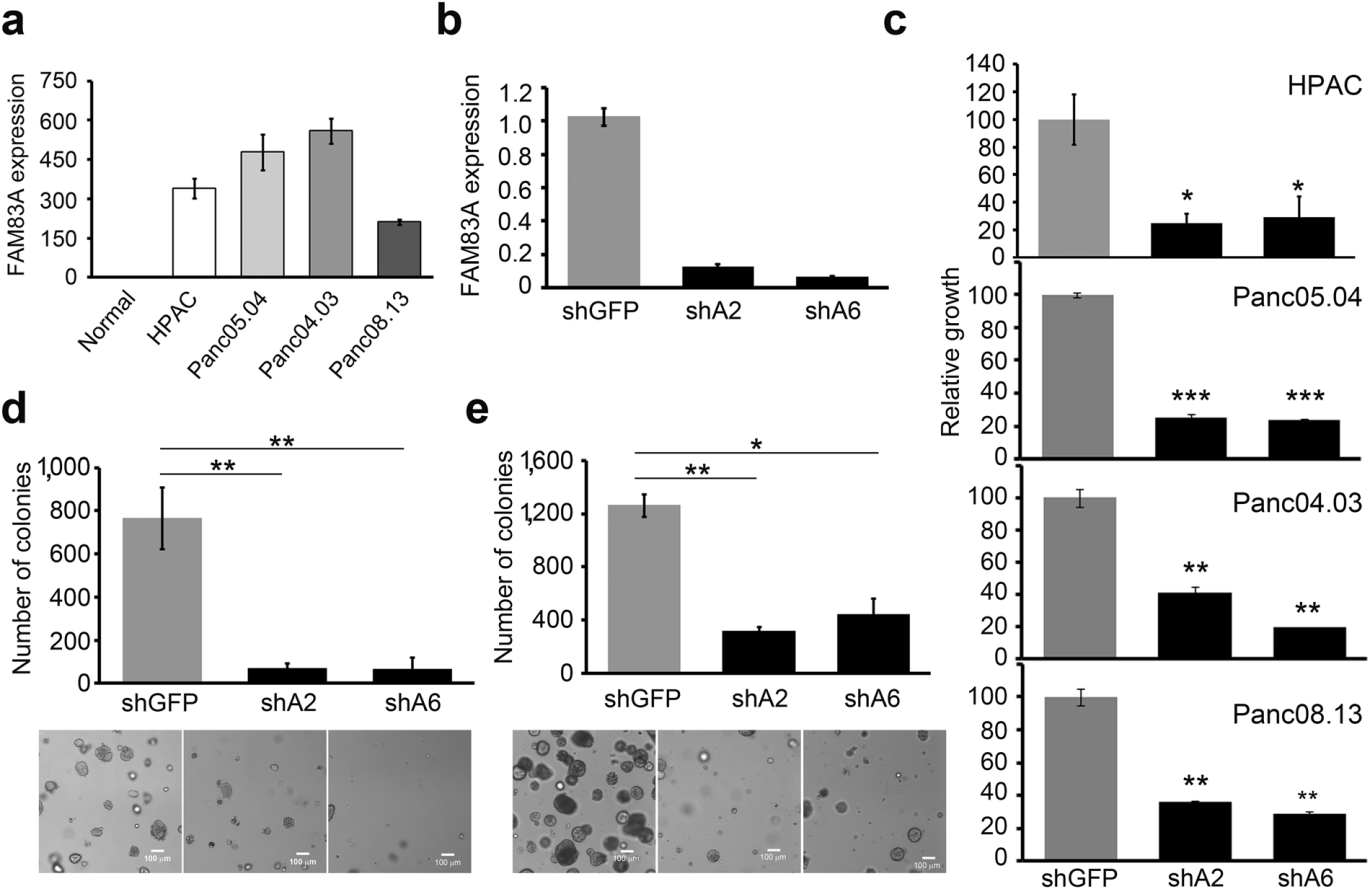
Elevated FAM83A is essential for the growth of pancreatic cancer cells. (**a**) *FAM83A* mRNA expression was assessed in a panel of pancreatic cancer cell lines compared to normal pancreas. (**b**–**e**) Ablation of FAM83A (**b**) using two different lentiviral shRNA constructs targeting FAM83A (shA2, shA6) drastically reduced the growth of pancreatic cancer cell lines in culture (**c**) and of HPAC cells in soft agar (**d**) and 3D organotypic matrigel cultures (**e**). All data are representative of three independent experiments (n = 3). All bar graphs show mean ± SD. Student’s t-test was used to assess statistical significance with *, ** and *** representing p < 0.05, p < 0.01 and p < 0.001 respectively.

Analysis of gene expression datasets^20^ identified that *FAM83A* expression is upregulated in human mammary epithelial cells (HMEC) expressing exogenous mutant *RAS*, but not other oncogenes such as c-sarcoma proto-oncogene (*SRC)*, c-Myc (*MYC)* and β-catenin (Fig. 1a). To confirm this observation, FAM83A expression was assessed by quantitative real-time PCR (qPCR) of telomerase reverse transcriptase (hTERT)-immortalized human mammary epithelial-1(HME1) cells exogenously expressing mutant Harvey Rat sarcoma (*HRAS)*, Neuroblastoma Rat Sarcoma *(NRAS) and KRAS*. All three *RAS* isoforms increased *FAM83A* gene expression >2.5 fold relative to the control (Fig. 1b). Given that the vast majority of PDAC are driven by oncogenic, mutant KRAS, we surveyed FAM83A expression in PDAC specimens using publically available The Cancer Genome Atlas (TCGA) and University of Texas Southwestern (UTSW) datasets. In three independent studies^21–23^, *FAM83A* gene expression was significantly elevated in pancreatic tumour tissue as compared to their associated-normal counterparts (Fig. 1c), and 14% of pancreatic cancers exhibit FAM83A gene amplification^24,25^. In three separate patient-derived pancreatic tumour samples, *FAM83A* expression was elevated compared to matched tumour-associated normal pancreas tissue (Supplementary Fig S1). In addition, pancreatic cancer cell lines exhibit increased expression of *FAM83A* when compared to the normal human pancreas (Fig. 2a). In contrast, *FAM83B*, another FAM83 member, was not elevated in the pancreatic cancer cell lines (Supplementary Fig. S1). Taken together, these data point at a selective increase in FAM83A expression in pancreatic cancers.

### FAM83A is essential for the growth and tumorigenicity of pancreatic cancer cells

**Figure 3:**
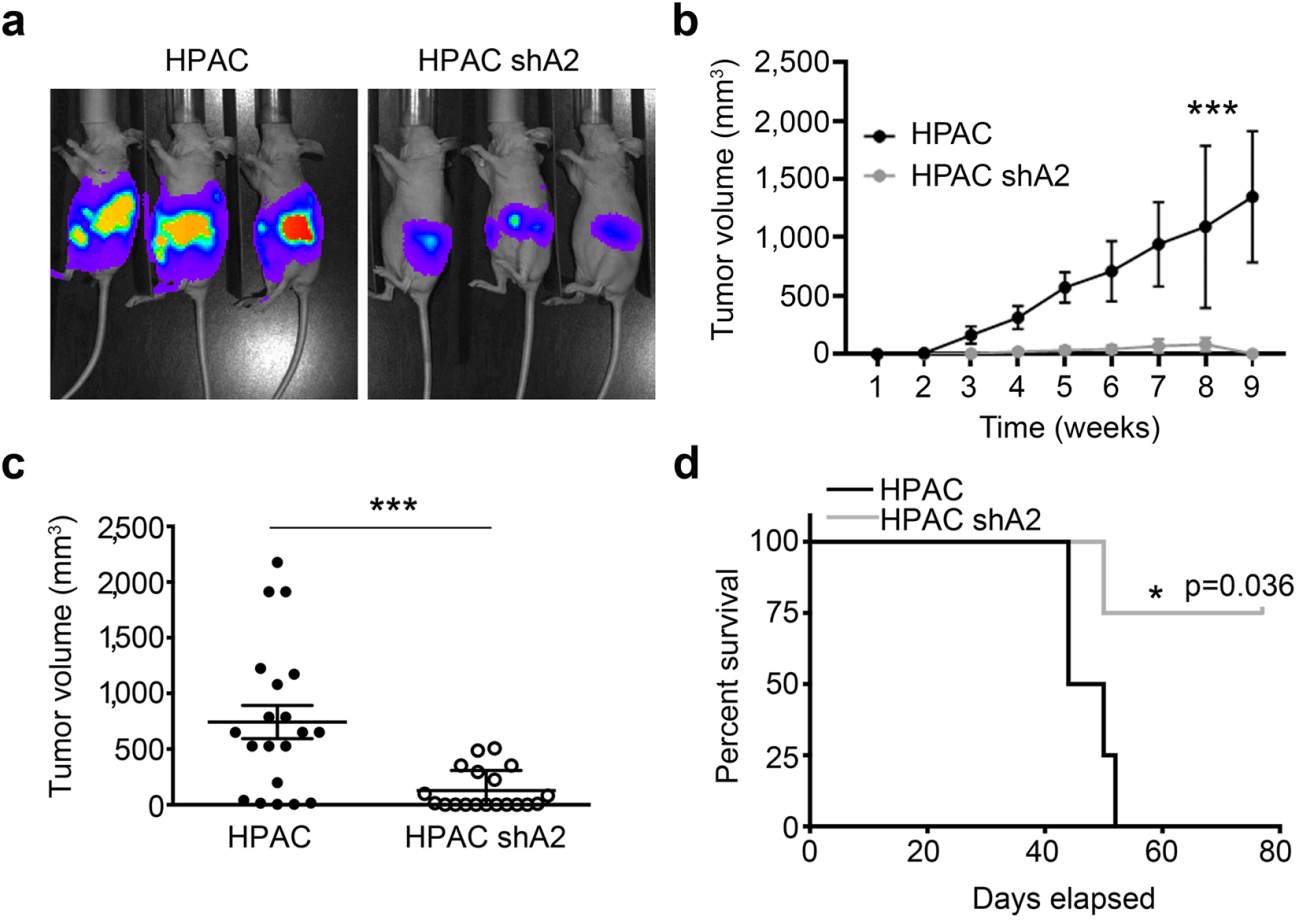
FAM83A ablation reduces tumorigenicity *in vivo*. HPAC-shA2 cells and control HPAC-shGFP cells (expressing GFP-LUC fusion protein) were injected orthotopically into the pancreas of immunocompromised mice. Bioluminescence images at day 21 post inoculation (**a**), tumor growth curve (**b**), tumour volume at the time of excision (**c**) and overall survival of mice (**d**) are shown. (**a**,**d**) are representative. (**b**) (mean ± SEM) and (**c**) (mean ± SD) are composite of three independent experiments (total n = 19). For panel b, statistical significance with a p value < 0.0001 was determined using two-way ANOVA (mixed-effects model; alpha 0.05) analysis. For panel c, student’s t-test was used to assess statistical significance with *** representing p < 0.001.

The mitogen activated protein kinase (MAPK) and PI3K signalling pathways downstream of KRAS are major contributors to pancreatic carcinogenesis. Importantly, FAM83A was originally identified as a positive regulator of MAPK and PI3K-signaling pathways^19^. Therefore, we hypothesized that the KRAS-mediated induction of *FAM83A* described above, creates a positive, feed-forward loop whereby FAM83A increases KRAS effector signalling, which functions to drive increased transcription of the FAM83A gene, ultimately driving pancreatic cancer proliferation and survival. To test this hypothesis, lentiviral constructs expressing two different short hairpin RNA (shRNA) targeting *FAM83A* (shA2, shA6) or a control shRNA (shGreen Fluorescent Protein/shGFP) were used to knockdown *FAM83A* in pancreatic cancer cell lines (HPAC, Panc04.03, Panc05.04, Panc08.13). Indeed, suppression of FAM83A expression by either shA2 or shA6 (Fig. 2b) reduced cell number by ~70–80% in 2-dimensional culture (Fig. 2c), and strongly suppressed anchorage-independent growth in soft agar (Fig. 2d) and 3Dimensional (3D)-organoids (Fig. 2e). To determine if the impaired growth following *FAM83A* ablation had any physiological significance to *in vivo* pancreatic tumour formation, FAM83A was knocked down in luciferase-expressing HPAC cells and the cells were transplanted orthotopically into the pancreas of immunocompromised mice. Following FAM83A knockdown, pancreatic tumours were significantly smaller when measured by bioluminescence imaging (Fig. 3a) or tumour volume (Fig. 3b,c), resulting in delayed mortality (Fig. 3d). These data clearly demonstrate that FAM83A is an important contributor to *in vivo* pancreatic tumour growth and that targeting FAM83A or its function has the potential to enhance patient survival.

### Suppression of FAM83A induces apoptosis in pancreatic cancer cells

**Figure 4:**
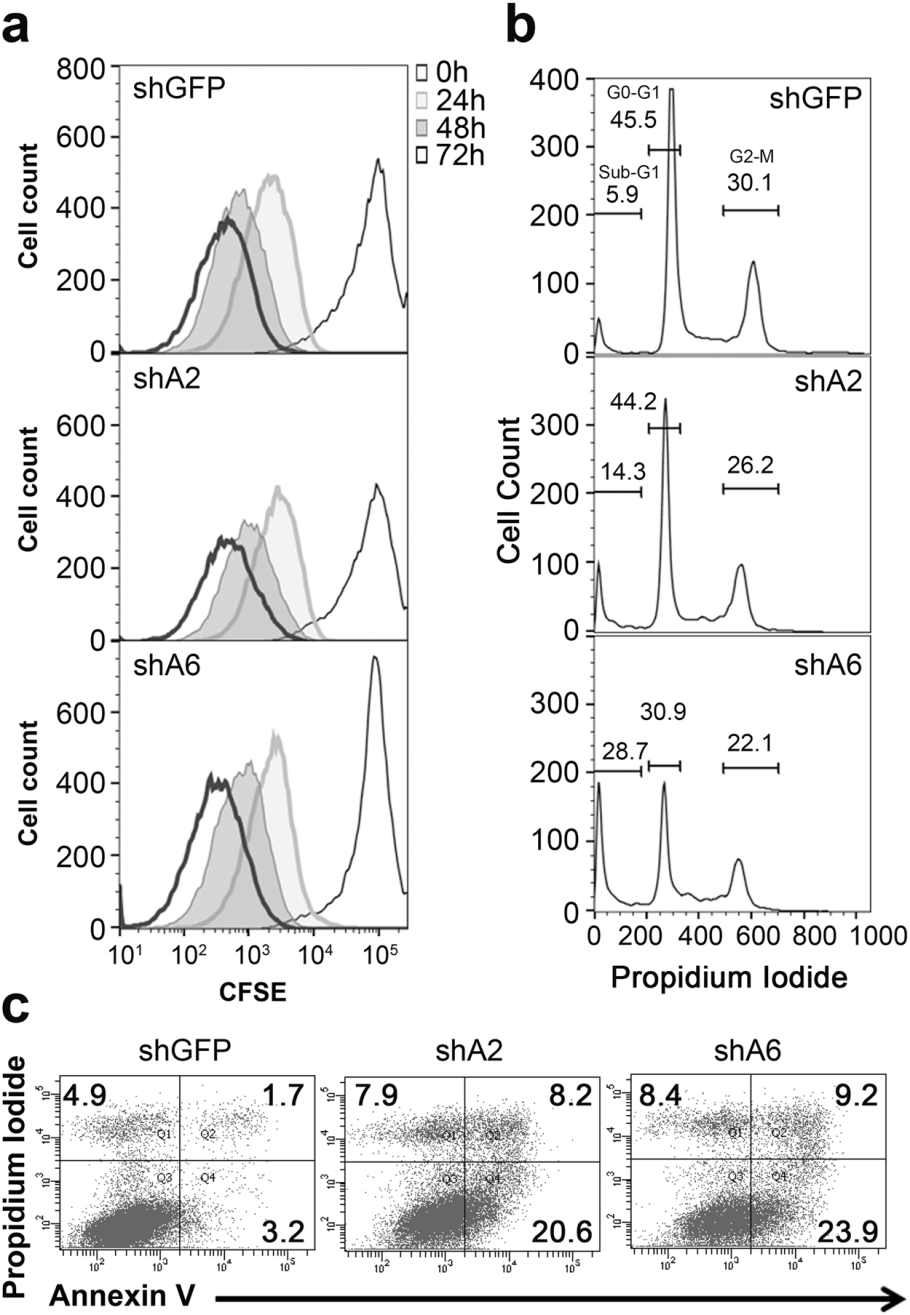
FAM83A loss induces apoptotic cell death. (**a**–**c**) HPAC-shGFP, HPAC-shA2 and HPAC-shA6 cells were assessed for proliferation and apoptosis over four days. Cells were labelled with CFSE and cellular proliferation was assessed by the dilution of CFSE at 0, 24, 48 and 72 h shown as overlaid histogram plots (**a**). Cells were stained with DNA-binding dye propidium iodide for cell cycle analysis by flow cytometry. The histogram plots in b show the sub-G1, G0-G1 and G2-M population at 96 h. (**c**) Cellular apoptosis was assessed by Annexin V and PI staining. The plots show increased numbers of apoptotic (Annexin V^+^) and dead (Annexin V^+^PI^+^) cells in the shA groups at 48 h. All data are representative of three independent experiments.

Since suppression of FAM83A resulted in severe growth impairment and inhibited tumorigenesis, we next sought to determine whether FAM83A loss impacted proliferative capacity or survival. First, proliferation was monitored using a membrane-protein labelling dye, 5(6)-carboxyfluorescein diacetate *N*-succinimidyl ester (CFSE) as a measure of cell division. CFSE labels the cells and is diluted with each cellular division resulting in distinct peaks when assessed by flow cytometry. FAM83A ablation did not alter the rate at which CFSE was reduced with each division, indicating that the proliferative capacity of cells lacking FAM83A is not impacted (Fig. 4a). Similar observations were made using the Bromodeoxyuridine (BrdU) incorporation assay, wherein the incorporation of the nucleoside analogue by actively proliferating PDAC cells was comparable in the presence and absence of FAM83A (Supplementary Fig. S2). Next, we assessed how FAM83A suppression impacted cell survival. Using propidium iodide (PI) staining and flow cytometry, *FAM83A* knockdown consistently increased the sub-G1 population to 14% (shA2) and 28% (shA6) when compared to 6% in control cells, indicating that FAM83A suppression results in enhanced cell death (Fig. 4b). Likewise, FAM83A knockdown increased the proportion of Annexin V-positive apoptotic cells from 4.9% to 28.8% (shA2) and 33.1% (shA6; Fig. 4c). Hence, the elevated levels of *FAM83A* observed in PDAC cells have a pro-survival function.

### FAM83A knockdown inhibits activation of the MEK-ERK survival signalling

**Figure 5:**
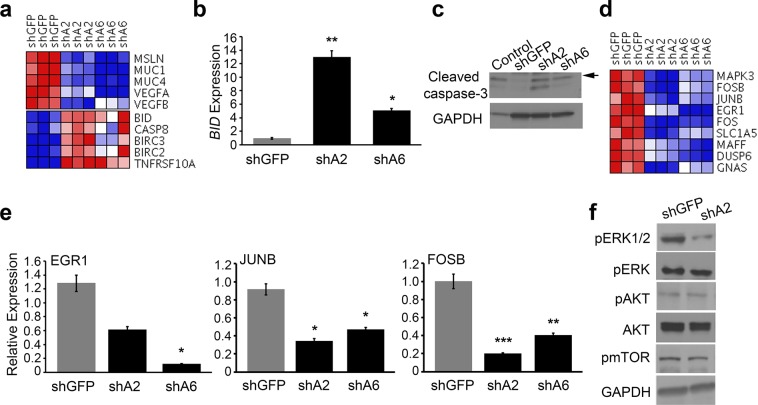
FAM83A promotes activation of pro-survival MEK/ERK signalling. RNA sequencing analysis of RNA extracted from HPAC-shGFP, HPAC-shA2 and HPAC-shA6 cells shown as heatmaps (**a**) indicated reduced expression of poor prognosis markers of pancreatic cancer and increased expression of pro-apoptotic genes. Data from each triplicate sample are shown. (**b**) qRT-PCR for pro-apoptotic Bid and immunoblotting for cleaved caspase-3 at 96 h (**c**). The control in (**c**) is lysate from HPAC cells treated with etoposide to induce cleaved caspase-3. (**d**) Pro-survival gene signature shown as heatmap was extracted from the RNA sequencing experiment described in (**a**). (**e**) qRT-PCR analysis for ERK- transcriptional targets *EGR1*, *JUNB* and *FOSB*. Significance values shown are relative to the shGFP control group. (**f**) Activation of MEK and PI3K pathways were assessed in HPAC-shGFP and HPAC-shA2 cells by measuring phosphorylation of ERK, mTOR and AKT. GAPDH was used as control. Data in C and F are representative of three independent experiments. Student’s t-test (two-tailed, unequal variance) was used to assess statistical significance with *, ** and *** representing p < 0.05, p < 0.01 and p < 0.001 respectively.

To understand how FAM83A contributes to survival signalling in pancreatic cancers, next generation RNA-sequencing (RNA-seq) analysis was performed to examine global gene expression changes in PDAC cells following FAM83A knockdown. Specifically, the knockdown of FAM83A resulted in 542 messengerRNAs that become differentially expressed (≥2 fold change, p < 0.05) (FAM83A knockdown gene signature and gene set enrichment analysis are shown in Supplementary Fig. S3). Importantly, FAM83A suppression clearly decreased the expression of hallmark genes indicative of aggressive pancreatic cancers such as Mesothelin, VEGF, MUC1 and MUC4 (Fig. 5a)^26^. Importantly, FAM83A knockdown upregulated genes associated with apoptosis and cell death, including pro-apoptotic gene caspase-8 (*CASP8)* and BH3 interacting-domain death agonist (*BID)* (Fig. 5a,b). BID and Caspase 8 are known regulators of apoptosis that trigger the release of mitochondrial proteins and activate effector caspases like caspase-3^27^. Accordingly, knockdown of FAM83A resulted in increased levels of cleaved caspase-3 comparable to the etoposide-treated positive control (Fig. 5c). The MEK/ERK pathway is known to protect cells from stress-induced caspase-8/BID cleavage^28^. Notably, FAM83A knockdown also reduced MEK/ERK-responsive genes, including *EGR1*, *FOSB and JUNB* (Figs. 5d,e). Early growth response protein 1 (*EGR1)* and the AP-1 components, *FOSB and JUNB* are known to regulate transcription in response to active MEK/ERK signalling, resulting in enhanced cell growth and survival^29,30^. Likewise, suppression of FAM83A reduced MEK-mediated ERK phosphorylation, but did not impact signalling to AKT and mTOR (Fig. 5f), indicating that FAM83A-mediated MEK/ERK activation is critical for the survival of PDAC cells.

**Figure 6:**
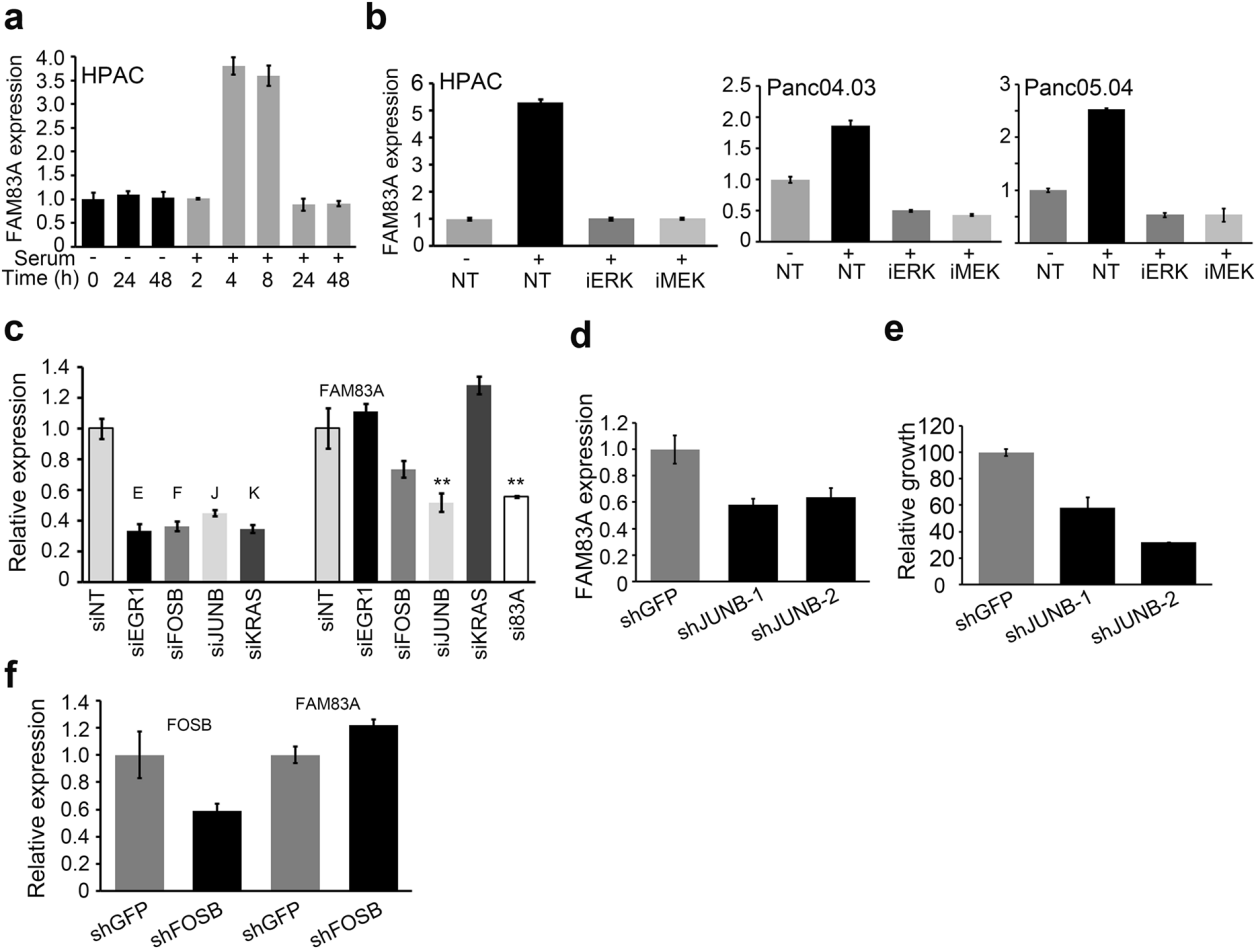
Elevated FAM83A expression is dependent on MEK/ERK signalling-driven JUNB transcription. (**a**) FAM83A mRNA expression induced upon serum stimulation of HPAC cells as assessed by qRT-PCR at the indicated times. (**b**) HPAC, Panc04.03 and Panc05.04 cells were pretreated with MEK and ERK pathway inhibitors prior to 4 h of serum stimulation to assess for induced *FAM83A* expression. (**c**) HPAC cells transfected with siRNA smartpools targeting EGR1, FOSB, JUNB, KRAS, FAM83A and a non-targeting (NT) control were assessed for serum-induced *FAM83A* expression. The individual knockdowns are shown on the left and identified as E (EGR1), F (FOSB), J (JUNB) and K (KRAS). *FAM83A* expression in each knockdown group is shown on the right together with the FAM83A knockdown (si83A). Student’s t-test was used to assess statistical significance with ** representing p < 0.01. (**d**–**e**) HPAC cells infected with lentivirus encoding two different shRNAs targeting JUNB (shJUNB1, shJUNB2) and control (shGFP) were assessed for serum-induced *FAM83A* expression (**d**) and growth (**e**). HPAC cells infected with lentivirus encoding shRNAs targeting FOSB (shFOSB) and control (shGFP) were assessed for *FOSB* and serum-induced *FAM83A* expression (**f**). All data are representative of three independent experiments.

We observed that FAM83A expression was strongly induced by >4-fold upon serum stimulation (Fig. 6a), and inhibition of either MEK or ERK blocked serum-induced *FAM83A* expression (Fig. 6b). As *EGR1* (E), *FOSB* (F) and *JUNB* (J) are MEK/ERK regulated transcription factors known to bind serum responsive elements (SREs), we used SMARTpool small interfering RNA (siRNA) targeting each in order to examine their importance in regulating FAM83A expression. While loss of *JUNB* significantly reduced serum-induced *FAM83A* expression, the loss of *FOSB* and *EGR1* resulted in slight or no reduction in *FAM83A*, respectively (Fig. 6c). We also confirmed these data using two different shRNAs against JUNB (shJunB-1 and shJUNB-2; Fig. 6d). Knockdown of JUNB significantly reduced cell growth phenocopying the loss of FAM83A (Fig. 6e). shRNA-mediated knockdown of FOSB failed to reduce FAM83A expression (Fig. 6f). Surprisingly FAM83A was not directly regulated by *KRAS*, as depletion of *KRAS* gene expression using two different lentiviral shRNAs (shKRAS#1 and shKRAS#2) and siRNA did not alter *FAM83A* expression (Supplementary Fig. S4A; Fig. 6c). Taken together, the MEK/ERK-regulated AP-1 transcription factor JUNB controls FAM83A expression in PDAC cells. FAM83A knockdown in turn strongly inhibits MEK/ERK activation and transcription of JUNB as shown in Fig. 5. Our data suggests the existence of a novel positive feed-forward loop involving MEK-ERK-JUNB-FAM83A that promotes pancreatic tumorigenesis.

### Murine KRAS-G12D p53-R172H cells require sustained FAM83A expression for tumor formation

**Figure 7:**
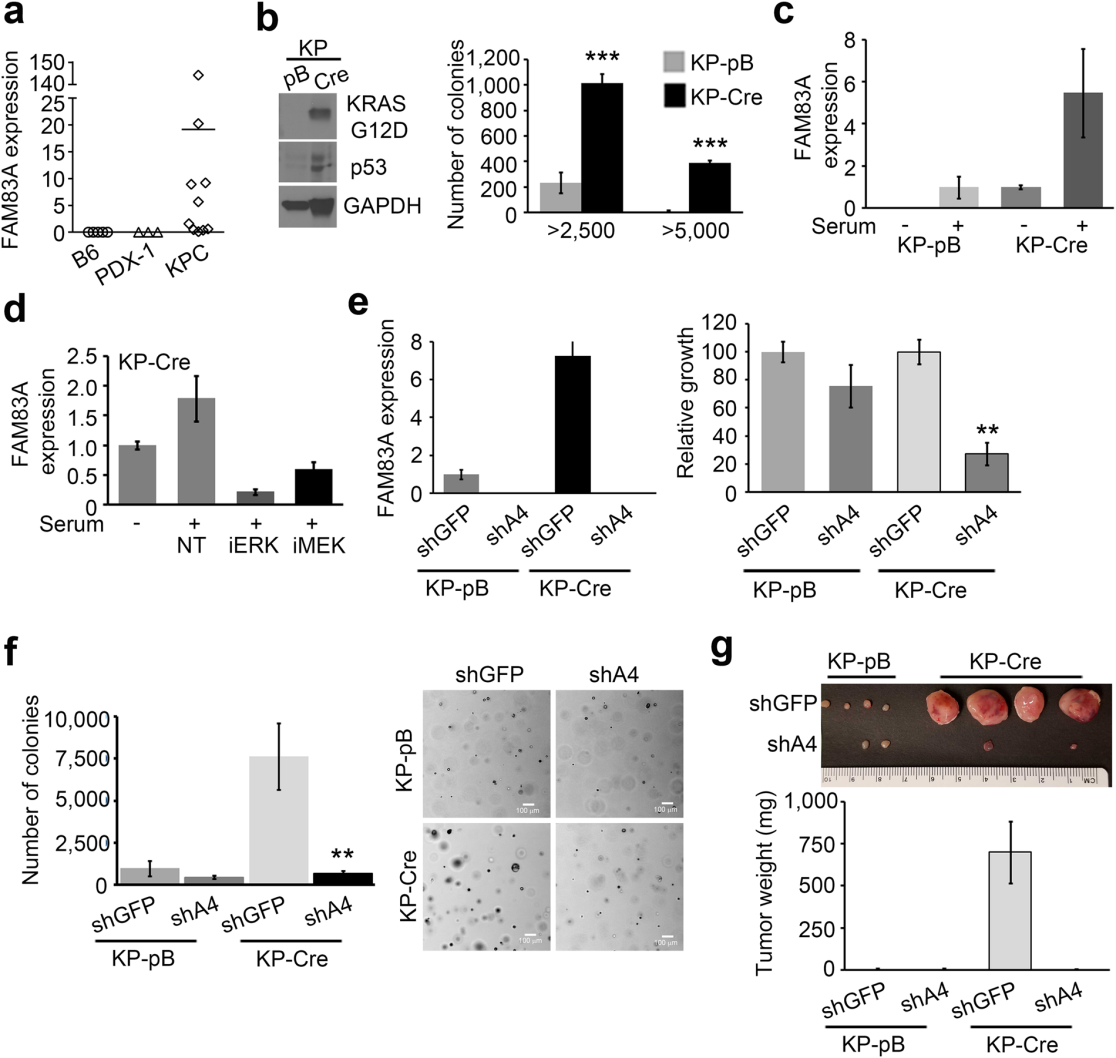
Elevated FAM83A expression is essential for growth and tumorigenicity of murine KPC cells. (**a**) FAM83A mRNA expression in spontaneous pancreatic tumours of LSL-KRAS-G12D LSL-p53-R172H Pdx1-Cre (KPC) transgenic mice (n = 10) compared to normal murine pancreas from C57BL/6 (n = 5) and control Pdx1-Cre (n = 3) mice. Statistical significance of p < 0.01 as per the unpaired Mann-Whitney test. (**b**) Pancreatic ductal epithelial cells isolated from the pancreas of LSL-KRAS-G12D LSL-p53-R172H (KP) mice were infected *in vitro* with retroviruses encoding Cre or control vector (pB). Immunoblotting for mutant KRAS and p53 confirmed generation of KP-Cre cells *in vitro* and anchorage-independent growth in soft agar was assessed as indication of transformation. *FAM83A* expression in control KP-pB and KP-Cre cells upon 24 h serum stimulation (**c**) and in KP-Cre cells upon serum stimulation following pre-treatment with MEK and ERK inhibitors (**d**). (**e**–**g**) KP-pB and KP-Cre cells were subjected to FAM83A knockdown using lentivirus encoding shRNA targeting mouse FAM83A (shA4) or control (shGFP) and assessed for *FAM83A* expression by qRT-PCR (**e**; left), growth in culture (e; right). KP-pB and KP-Cre cells with FAM83A knockdown were also assessed for anchorage independent growth in soft agar (**f**) and for *in vivo* tumorigenicity upon subcutaneous injections (n = 4) in the flanks of immunocompromised mice (**g**). Images of the tumours were from day 21 post inoculation. All data in (**b**–**g**) are representative of two independent experiments. All bar graphs show mean ± SD. Student’s t-test was used to assess statistical significance with ** and *** representing p < 0.01 and p < 0.001 respectively.

Murine pancreatic cancer models such as the KPC (KRAS-G12D p53-R172H Pdx1-Cre) mice offer a critical resource for the testing of potential therapeutic targets^31^. As observed in human pancreatic tumours and tumour cell lines, FAM83A expression was significantly elevated in pancreatic tumours isolated from the KPC mice (Fig. 7a). Moreover, pancreatic epithelial cells from the LSL-KRAS-G12D LSL-p53-R172H (KP) mice transformed *in vitro* by infection with retroviral particles encoding Cre recombinase (KP-Cre) also expressed higher *FAM83A* levels relative to the control KP-pB cells (infected with control pBabe vector-expressing retrovirus; Fig. 7c). Expression of the mutant KRAS protein and stable p53 protein were confirmed in the Cre-transformed cells (KP-Cre) along with their ability to grow anchorage independently in soft agar (Fig. 7b). Similar to our results with the human PDAC cells, inhibitors to both MEK and ERK significantly inhibited FAM83A expression in the KP-Cre cells (Fig. 7d). Targeting FAM83A expression in the KP-Cre cells using shRNA against murine FAM83A (shA4) significantly reduced *in vitro* cell growth (Fig. 7e), anchorage-independent growth in soft agar (Fig. 7f) and *in vivo* tumour formation (Fig. 7g).

**Figure 8:**
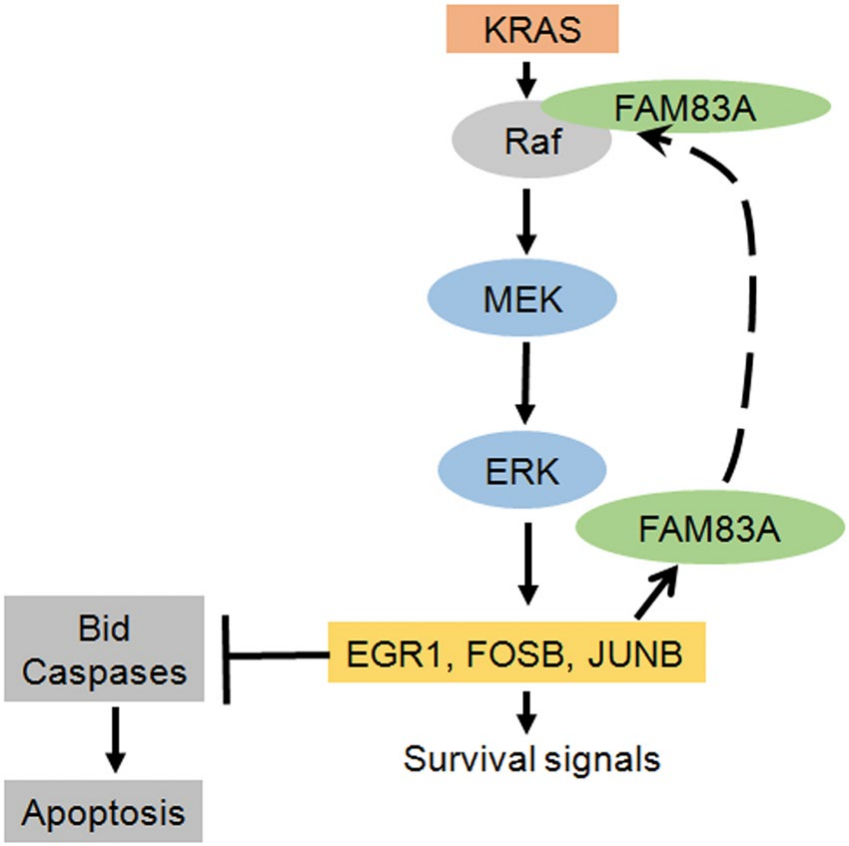
Elevated FAM83A expression in pancreatic cancers is driven by a MEK/ERK-JUNB-driven feed-forward signalling loop. The oncogene FAM83A promotes activation of pro-survival MEK-ERK signalling pathway that regulates transcription of JUNB which feeds forward to maintain elevated expression of FAM83A in turn promoting pancreatic cancer growth, survival and tumorigenicity. Disruption of the MEK-FAM83A positive loop will serve as a tumour-specific therapeutic target in KRAS-dependent and independent pancreatic cancers.

In summary, we have identified a MEK/ERK-AP1-FAM83A positive feed-back loop that promotes pancreatic cancer growth and tumorigenesis (Fig. 8). Dismantling the positive feed-back loop by targeting FAM83A expression or function would block essential MEK/ERK survival signalling and the MEK/ERK-regulated transcriptional activation in a tumour-specific manner. This strategy will have the additional advantage of a wider therapeutic window and lower side effect/toxicity to normal non-tumour cells.

## Discussion

Identifying the genetic drivers responsible for carcinogenesis will help uncover molecular targets for future cancer therapies, screen for individuals at risk, and predict a patient’s response to cancer therapies. We demonstrate here that the oncogene FAM83A is upregulated in pancreatic cancer cells, and functions to promote cancer cell survival. Importantly, we identify a MEK/ERK-FAM83A feed-forward signalling loop as the driver of oncogenic FAM83A expression and propose that patients with elevated FAM83A may benefit from therapeutic targeting of the MEK/ERK-FAM83A axis.

The FAM83 family was identified based on its transforming potential and ability to induce resistance to EGFR TKIs, solidifying its role in EGFR/MAPK signaling^15,19^. Since our initial discoveries, numerous reports have identified roles for various FAM83 proteins in cancer^32–34^. Moreover, genomics analyses have identified consistent upregulation of FAM83 member expression across the majority of tumour types, with high levels of FAM83 gene expression frequently associated with poor prognosis^18^. The smallest FAM83 member and focus of the current study, FAM83A, is not frequently mutated in cancer (except in skin cutaneous melanoma)^24,25^. Rather, it is overexpressed in lung, breast, ovary, colon and pancreas cancers. Beyond simply being overexpressed, FAM83A expression is an unfavourable prognostic gene in lung cancers^35,36^, and was documented as a prognostic biomarker (together with Keratin-19, Squamous carcinoma antigen (SCCA) and other proteins) capable of identifying circulating breast and lung cancer cells that predicted for shorter patient survival time^37^. More recently, Snijders *et al*. used a multi-omics approach to reveal increased expression of a FAM83 gene family was predictive of poor survival in many human cancers including pancreatic cancer^18^. Consistent with our findings, Chen *et al*. show that FAM83A expression is elevated in PDAC patients and high expression correlates with significantly poor overall and disease free survival, due in part to the gain of cancer stem cell features and chemoresistance to Gemcitabine^38^.

While little is known about what regulates FAM83A expression, it is clear that when overexpressed, FAM83A can lead to aberrant activation of MEK/ERK signalling through its interaction with C-RAF^16^, by potentially stabilizing signalling complexes. In publically available gene expression data^23^, elevated FAM83A expression correlates with an increased MAPK gene expression signature (Supplementary Fig. S5). While the impact of FAM83A expression on MEK/ERK signalling is of interest, even more intriguing is our finding that FAM83A gene expression is positively regulated by the AP1 transcription factors JUNB and FOSB downstream of MEK/ERK signalling. Further promoter-binding studies are needed to confirm the direct role of JUNB and the identity of its partners. However, the consequence of this regulatory cascade is that, increased MEK/ERK activity increases FAM83A expression, which further increases MEK/ERK activity in a positive feed-forward signalling loop. Importantly, as shown here, suppression of FAM83A expression in human or murine pancreatic cancer cells can break this feed-forward loop, induce apoptosis, and thereby reduce the size of pancreatic tumours. Similar findings have been reported in human epidermal growth factor receptor 2 (Her2)-positive as well as triple negative breast cancer, both of which are aggressive tumour sub-types^16,39^. We therefore propose that, interfering with this positive signalling loop by inhibiting FAM83A expression or function could impact pancreatic cancer cell growth and survival.

Importantly, the novel MEK/ERK-FAM83A self-sustaining loop described here may be independent of mutant KRAS expression, as knockdown of KRAS did not affect FAM83A expression, likely due to continued MEK/ERK activation independent of mutant KRAS. One possibility is the contribution of the remaining HRAS and NRAS. Hayes *et al*. reported enduring activation of ERK signalling in pancreatic cancer cells despite KRAS knockdown, reaffirming that in some pancreatic cancer cells, MEK/ERK activation is independent of KRAS^40^. In KRAS-independent pancreatic tumours, the continued activation of MEK/ERK signalling by other mechanisms may still drive the MEK-FAM83A positive feed-back loop. A recent kinome siRNA screen identified a remarkable diversity of kinases that contributed to resistance to MEK/ERK pathway inhibition^41^. These findings suggest that once cells are transformed, they can evolve a striking heterogeneity in proliferative and pro-survival signalling. For example, as in many other cancers, amplification and overexpression of EGFR activates oncogenic MEK and PI3K signalling in pancreatic cancers, even those harbouring mutant RAS^42^. Although the EGFR inhibitor Erlotinib has modestly improved patient survival, acquired resistance to this drug remains a major stumbling block. Given that elevated FAM83A expression results in acquired resistance to EGFR TKIs^19^, FAM83A expression may help predict the response to EGFR inhibitor therapy in patients with pancreatic cancer.

Interestingly, FAM83A was also reported to be one of the highly tyrosine-phosphorylated proteins in Trastuzumab-resistant HER2-positive breast cancer cells, and again, FAM83A ablation suppressed the growth of Trastuzumab-resistant cells^43^. The possibility that FAM83A acts as a scaffold, promoting or stabilizing oncogenic avian erythroblastosis oncogene B (ErbB)-MAPK signalling pathways and that its function may be regulated upon phosphorylation by the ErbB family of receptors such as EGFR and HER2 opens up novel targeting avenues. Moreover, we postulate that the MEK/ERK-FAM83A feed-forward loop may be a novel therapeutic target in pancreatic cancer cells, including those that remain dependent on mutant RAS, as well as those that are independent of mutant RAS.

FAM83A is mostly undetectable in normal tissues, thus, tumours with elevated FAM83A expression are postulated to have a wide therapeutic window^17^. Finding methods to suppress FAM83A expression or prevent FAM83A from serving as a signalling scaffold are leading potential therapeutic approaches. Recently, small molecules have been successfully designed to block protein-adaptor interactions in the RAS/RAF pathway such as Rocaglamide blocking prohibitin/C-RAF^44^ and Rigosertib blocking RAS-RAF interaction^13^. Inhibitors or peptides to weaken the FAM83A/C-RAF interactions or the FAM83A/EGFR interaction by specifically targeting the amino-terminal DUF1669 responsible for the signalling interactions are potential avenues to directly target FAM83A function. Another feasible option is employing nanoparticles encapsulating siRNA targeting oncogenic FAM83A mRNA. Diverse kinds of non-immunogenic nanoparticles with increased *in vivo* stability and armed with tumour-homing peptides are currently in clinical trials for targeting oncogenic mRNAs in many solid tumors^45–47^. The means of targeting oncogenic FAM83A in pancreatic cancers may still be elusive but it is very likely to overcome current therapeutic roadblocks with much less harm to normal healthy cells.

## Materials and Methods

### Mice

LSL KRAS G12D (01XJ6)^48^, p53 LSL R172H (01XAF)^49^ and Pdx-1-Cre/+(01XL5)^50^ mice were obtained from National Cancer Institute mouse repository (Frederick, MD, USA) and bred in our animal facility to generate the KRAS G12D p53 R172H (KP) and KRAS G12D p53 R172H PDX-1-Cre (KPC) mice. Genotyping was routinely performed by Transnetyx (Cordova, TN, USA) using real-time Polymerase Chain Reaction (PCR).

### Cell culture

All PDAC cell lines were obtained from ATCC (Manassas, VA, USA) and grown in a humidified atmosphere containing 5% CO_2_ at 37 °C. HPAC, Panc 04.03, Panc 08.13 and Panc 05.04 cells were grown in medium described previously^51^. Cells were used from frozen stocks within 10 passages upon receipt from ATCC. Cells in culture were routinely tested for mycoplasma using the MycoAlert Detection kit (LT07-218; Lonza, Basel, Switzerland). Normal pancreatic tissue was obtained from a pancreatic resection biopsy and the tissue identified as normal by the pathologist. All human tissue samples were obtained under discarded tissue protocol (IRB number CASE12Z12) in full accordance with the guidelines and regulations approved by Case Western Reserve University Institutional Review Board. The institutional review board waived informed consent for this discarded tissue protocol.

For pancreatic ductal epithelial cell cultures, pancreas removed from KRAS G12D p53 R172H (KP) mice were minced with scalpels and digested in digestion medium containing DMEM/F-12, Penicillin/Streptomycin (#30-009-CI; Corning), Collagenase (#C0130; Sigma Aldrich), Hyaluronidase (#H3506; Sigma Aldrich), Insulin, FCS and Polymyxin-B sulphate (#P4932; Sigma Aldrich) for 20 min at 37 °C. The digested solution was spun at 300 g for 5 min and pellet resuspended in M87 culture medium (composition as described in^52^). This suspension was filtered through 100 μ filter and the filtrate was passed through 40 μ filter. The ductal organoids stuck on the 40 μ filter were washed and collected in a fresh tube and spun at 300 g for 5 min. KP ductal organoids were grown on Corning^TM^ Biocoat^TM^ Collagen I-coated multiwell plates in M87 medium. The cells that grew out onto the dishes were trypsinized and passaged in culture as needed.

The MEK Inhibitor (U0126; #70970) and PI3K inhibitor (LY294002; #70920) were both used at 50 μM and obtained from Cayman Chemicals (Ann Arbor, MI, USA); ERK inhibitor used at 10 μM (SCH772984; #S7101) was from Selleck Chemicals (Houston, TX, USA). Cells were pretreated for 2 h with the inhibitors prior to use in experiments. For FAM83A expression, cells serum starved for 16 h were stimulated with 10% serum for 4 h following which cells were harvested for RNA extraction.

### Short hairpin RNA and Small interfering RNA mediated knockdown

Lentiviruses were packaged and used to infect target cells as previously described^53^. Briefly, cells infected overnight with titered lentiviruses were rested for a day, reinfected and rested prior to puromycin selection for 48 h and used in experiments 6 days after transduction. PLK0.1 vectors containing shRNAs targeting human FAM83A (sh83A2 TRC Version: TRCN0000168628; Clone Name NM_032899.4-2327s1c1 and sh83A6 TRCN0000168368: Clone Name: NM_032899.4-1008s1c1), KRAS (TRC Version: TRCN0000033259; Clone Name: NM_033360.2-4328s1c1 and TRC Version:TRCN0000033262; Clone Name: NM_033360.2-509s1c1), JUNB (shJUNB-1 TRC Version: TRCN0000232087**;** Clone Name: NM_002229.2-1536s21c1 and shJUNB-2 TRC Version: TRCN000014944**;** Clone Name: NM_002229.2-1054s1c1), FOSB (shFOSB TRC Version: TRCN0000424014; Clone Name: NM_006732.2-1767s21c1) and mouse FAM83A (shA4 TRC Version: TRCN0000264557**;** Clone Name: NM_173862.2-777s21c1and TRC Version: TRCN00000264555**;** Clone Name: NM_173862.2-1577s21c1) were purchased from Sigma Aldrich. PLK0.1 vector containing shRNA-targeting GFP has been previously described^53^. pBabe-puro-control and pBAbe-puro-cre have been described previously^15^. Retroviruses were generated using protocol previously described^54–56^. KP murine pancreatic ductal epithelial cells were transduced with retroviral particles encoding either pBABE-puro-control or pBabe-puro-cre. The Dharmacon (Lafayette, CO, USA) ON-TARGET plus Human FAM83A (84985); KRAS (3845); EGR1 (1958); FOSB (2354); JUNB (3726) siRNA SMARTpools, were obtained from GE Healthcare. siRNA transfections were done as per manufacturer’s instructions for Lipofectamine RNAiMAX (#13778-075; Invitrogen, Thermo Fisher Scientific, Waltham, MA, USA) and siRNA transfected cells were used for experiments 24 h post-tranfection. pFLUG-GFP-LUC (Firefly) fusion construct used to build cells for bioluminescence measurement has been described previously^51,57^.

### Growth assays and three dimensional organotypic cultures

For growth assays, 25 000 cells/well were plated in six-well plates (in triplicates) for the indicated days and total cell number for each well was quantified using a Beckman Coulter counter (Beckman Coulter, Brea, CA, USA). Average cell number per well for each triplicate on day 4 is shown. For three-dimensional cultures, soft agar assays and organotypic culture in matrigel were as described previously^54,56^. All data presented are representative of three independent, biological replicates. Error bars represent standard deviation.

### Flow cytometry, cell proliferation and apoptosis assays

All assays used HPAC cells 6 days after transduction with lentiviral particles expressing shFAM83A. 1–2 × 10^6^ HPAC cells were labelled with 1 μM CFSE (#21888; Sigma) for 15 min at 37 °C, washed well with 1x PBS and cultured in replicates of 1 × 10^5^ cells that were monitored for CFSE dilution by flow cytometry over 5–7 days. For BrdU assays, cells were labelled in culture with BrdU at 37 °C for 3 h and incorporation measured by staining with anti-BrdU FITC antibody as per manufacturer’s protocol for BD Pharmingen BrdU FITC flow kit (#51–2354AK, San Jose, CA, USA).

For apoptosis, Annexin V FITC staining kit (#88-8005-72) from eBiosciences (San Diego, CA, USA) was used. Briefly 2–5 × 10^5^ cells in 1x binding buffer were stained with 5 μl Annexin V FITC for 10 min. Cells were washed thoroughly and stained with 5 μl of Propidium Iodide for immediate analysis by flow cytometry.

For cell cycle analysis, cells were washed with PBS, fixed with cold 70% ethanol for an hour at −20 °C. Fixed cells were washed with PBS and treated with 20 μg/ml of RNase A for 30 min at 37 °C. Cells on ice were stained with 50 μg/ml of Propidium Iodide for 60 min prior to flow cytometry. BD Biosciences LSR II was used for CFSE and BrdU analysis and Attune NxT (Thermo Fisher Scientific) was used for the cell cycle analysis. Data were analysed using FACSDiva software version 6.2 (BD Biosciences, San Jose, CA, USA) or FlowJo for Windows Version 10.1 (Ashland, OR, USA).

### Microscopy, western blot analysis, and quantitative real-time RT-PCR (qPCR)

Leica-DMI6000 AF inverted microscope (Leica Microsystems, Wetzlar, Germany) was used to capture images of colonies in soft agar and Metamorph (Molecular Devices, Sunnyvale, CA, USA) was used to quantify the colonies. Western blots were conducted as described previously^55,58^. Primary antibodies used were GAPDH (#CB1001; Calbiochem, EMD Millipore, Burlington, MA, USA), phosphorylated ERK Thr202/Tyr204 (#4370; Cell Signaling, Danvers, MA, USA), ERK (#4695; Cell Signaling), phosphorylated AKT (#4060; Cell Signaling), phosphorylated mTOR Ser2448 (#2971, Cell Signaling), RAS G12D Mutant (#14429 S; Cell Signaling) and p53 (#3396; Cell Signaling). Secondary antibodies used were HRP-linked Anti-Mouse (#7076; Cell Signaling) and HRP-linked Anti-Rabbit (#7074; Cell Signaling). For qRT-PCR, RNA isolation and gene expression analysis was as previously described^51^. Intron-spanning primers used for qRT-PCR are detailed in Supplementary Table S1. For FAM83A expression analysis, HPAC cells were serum starved for 16 h before stimulating with 10% serum for 4 h.

### Mouse xenografts

Athymic NCr (nude) mice were bred and maintained at the Athymic Animal and Xenograft Core facility at the Case Western Reserve University Case Comprehensive Cancer Center. For tumorigenicity assays, 1 × 10^6^ of HPAC-shGFP or HPAC-shA2 cells were resuspended in 50 μl of 1:1 mix of matrigel:media mix. Orthotopic pancreatic injections were performed on 8–12 week old male or female Nude mice as described^51^ previously. KPC-shGFP and KPC-shA2 cells were injected in 100 μl matrigel-media mix into the flanks of the mice. For all experiments, tumour volume was also measured manually using calipers and tumour weight assessed following euthanasia and tumour retrieval. All animals were used in compliance with the guidelines approved by the Case Western Reserve University Institutional Animal Care and Use Committee.

### RNA sequencing and analysis

Quality control of total RNA samples was executed using Qubit (Invitrogen) for quantification and Agilent 2100 Bioanalyzer (Agilent Technologies, Santa Clara, CA, USA) analysis to assess quality using a cut-off of RIN > 7.0 to select specimens for further analysis. For library preparation, the Illumina TruSeq Stranded Total RNA kit (Illumina Inc, San diego, CA, USA) with Ribo Zero Gold for rRNA removal was used. This protocol starts by using the Ribo-Zero kit to remove ribosomal RNA (rRNA) from 0.10–1 µg of Total RNA using a hybridization/bead capture procedure that selectively binds rRNA species using biotinylated capture probes. The resulting purified mRNA is used as input for the Illumina TruSeq kit in which libraries are tagged with unique adapter-indexes. Final libraries were validated on the Agilent 2100 Bioanalyzer, quantified via qPCR and pooled at equimolar ratios. Pooled libraries were diluted, denatured and loaded onto the Illumina HiSeq. 2500 using a paired-end Rapid Run flowcell. Following sequence alignment, the differentially expressed genes that are in common between the two distinct shRNAs were intersected and only annotated coding genes were selected. There were 542 differentially expressed genes that become differentially expressed ( ≥ 2 fold change, p < 0.05). Heatmaps of selected signature genes were generated using Hierarchical Clustering and Hierarchical ClusteringImage modules available from GenePattern (Broad Institute, University of California, San Diego, CA, USA). Gene set enrichment analysis was performed using the g:Profiler database (http://biit.cs.ut.ee/gprofiler/index.cgi)^59,60^.

### Data and statistical analysis

GraphPad Prism (La Jolla, CA, USA) and Microsoft Excel were used to plot data and Student’s t test or non-parametric Mann-Whitney test were used to calculate statistical significance.

## Supplementary information

Supplementary Information

## Data Availability

The datasets analysed during the current study are available from the corresponding author on reasonable request.

## References

[1] Howlader, N. *et al*. *SEER Cancer Statistics Review*, 1975–2014).

[2] Gaianigo Nicola, Melisi Davide, Carbone Carmine (2017). EMT and Treatment Resistance in Pancreatic Cancer. Cancers.

[3] Rhim AD (2012). EMT and dissemination precede pancreatic tumor formation. Cell.

[4] Smigiel Jacob, Parameswaran Neetha, Jackson Mark (2018). Targeting Pancreatic Cancer Cell Plasticity: The Latest in Therapeutics. Cancers.

[5] Rahib L (2014). Projecting cancer incidence and deaths to 2030: the unexpected burden of thyroid, liver, and pancreas cancers in the United States. Cancer Res.

[6] Kelley RK, Ko AH (2008). Erlotinib in the treatment of advanced pancreatic cancer. Biologics.

[7] Moore MJ (2007). Erlotinib plus gemcitabine compared with gemcitabine alone in patients with advanced pancreatic cancer: a phase III trial of the National Cancer Institute of Canada Clinical Trials Group. J Clin Oncol.

[8] Aichler M (2012). Origin of pancreatic ductal adenocarcinoma from atypical flat lesions: a comparative study in transgenic mice and human tissues. J Pathol.

[9] di Magliano MP, Logsdon CD (2013). Roles for KRAS in pancreatic tumor development and progression. Gastroenterology.

[10] Cox AD, Fesik SW, Kimmelman AC, Luo J, Der CJ (2014). Drugging the undruggable RAS: Mission possible?. Nat Rev Drug Discov.

[11] Zeitouni Daniel, Pylayeva-Gupta Yuliya, Der Channing, Bryant Kirsten (2016). KRAS Mutant Pancreatic Cancer: No Lone Path to an Effective Treatment. Cancers.

[12] Zimmermann G (2013). Small molecule inhibition of the KRAS-PDEdelta interaction impairs oncogenic KRAS signalling. Nature.

[13] Athuluri-Divakar SK (2016). A Small Molecule RAS-Mimetic Disrupts RAS Association with Effector Proteins to Block Signaling. Cell.

[14] Lito P, Solomon M, Li LS, Hansen R, Rosen N (2016). Allele-specific inhibitors inactivate mutant KRAS G12C by a trapping mechanism. Science.

[15] Cipriano R (2012). FAM83B mediates EGFR- and RAS-driven oncogenic transformation. J Clin Invest.

[16] Cipriano R (2014). Conserved oncogenic behavior of the FAM83 family regulates MAPK signaling in human cancer. Mol Cancer Res.

[17] Bartel CA, Parameswaran N, Cipriano R, Jackson MW (2016). FAM83 proteins: Fostering new interactions to drive oncogenic signaling and therapeutic resistance. Oncotarget.

[18] Snijders AM (2017). FAM83 family oncogenes are broadly involved in human cancers: an integrative multi-omics approach. Mol Oncol.

[19] Lee SY (2012). FAM83A confers EGFR-TKI resistance in breast cancer cells and in mice. J Clin Invest.

[20] Bild AH (2006). Oncogenic pathway signatures in human cancers as a guide to targeted therapies. Nature.

[21] Badea L, Herlea V, Dima SO, Dumitrascu T, Popescu I (2008). Combined gene expression analysis of whole-tissue and microdissected pancreatic ductal adenocarcinoma identifies genes specifically overexpressed in tumor epithelia. Hepatogastroenterology.

[22] Iacobuzio-Donahue CA (2003). Exploration of global gene expression patterns in pancreatic adenocarcinoma using cDNA microarrays. Am J Pathol.

[23] Pei H (2009). FKBP51 affects cancer cell response to chemotherapy by negatively regulating Akt. Cancer Cell.

[24] Cerami E (2012). The cBio cancer genomics portal: an open platform for exploring multidimensional cancer genomics data. Cancer Discov.

[25] Gao J (2013). Integrative analysis of complex cancer genomics and clinical profiles using the cBioPortal. Sci Signal.

[26] Iacobuzio-Donahue CA (2003). Highly expressed genes in pancreatic ductal adenocarcinomas: a comprehensive characterization and comparison of the transcription profiles obtained from three major technologies. Cancer Res.

[27] Kim WS (2017). The caspase-8/Bid/cytochrome c axis links signals from death receptors to mitochondrial reactive oxygen species production. Free Radic Biol Med.

[28] Shonai T (2002). MEK/ERK pathway protects ionizing radiation-induced loss of mitochondrial membrane potential and cell death in lymphocytic leukemia cells. Cell Death Differ.

[29] Hipskind RA, Baccarini M, Nordheim A (1994). Transient activation of RAF-1, MEK, and ERK2 coincides kinetically with ternary complex factor phosphorylation and immediate-early gene promoter activity *in vivo*. Mol Cell Biol.

[30] Vickers ER (2004). Ternary complex factor-serum response factor complex-regulated gene activity is required for cellular proliferation and inhibition of apoptotic cell death. Mol Cell Biol.

[31] Hingorani SR (2005). Trp53R172H and KrasG12D cooperate to promote chromosomal instability and widely metastatic pancreatic ductal adenocarcinoma in mice. Cancer Cell.

[32] Mao Y, Liu J, Zhang D, Li B (2016). miR-143 inhibits tumor progression by targeting FAM83F in esophageal squamous cell carcinoma. Tumour Biol.

[33] Shen CQ (2017). High Expression of FAM83B Predicts Poor Prognosis in Patients with Pancreatic Ductal Adenocarcinoma and Correlates with Cell Cycle and Cell Proliferation. J Cancer.

[34] Wang Z (2013). FAM83D promotes cell proliferation and motility by downregulating tumor suppressor gene FBXW7. Oncotarget.

[35] Li Y (2005). BJ-TSA-9, a novel human tumor-specific gene, has potential as a biomarker of lung cancer. Neoplasia.

[36] Liu L (2008). Detection of circulating cancer cells in lung cancer patients with a panel of marker genes. Biochem Biophys Res Commun.

[37] Liu L (2014). A rapid nested polymerase chain reaction method to detect circulating cancer cells in breast cancer patients using multiple marker genes. Oncol Lett.

[38] Chen S (2017). FAM83A is amplified and promotes cancer stem cell-like traits and chemoresistance in pancreatic cancer. Oncogenesis.

[39] Bartel CA, Jackson MW (2017). HER2-positive breast cancer cells expressing elevated FAM83A are sensitive to FAM83A loss. PLoS One.

[40] Hayes TK (2016). Long-Term ERK Inhibition in KRAS-Mutant Pancreatic Cancer Is Associated with MYC Degradation and Senescence-like Growth Suppression. Cancer Cell.

[41] Johnson GL, Stuhlmiller TJ, Angus SP, Zawistowski JS, Graves LM (2014). Molecular pathways: adaptive kinome reprogramming in response to targeted inhibition of the BRAF-MEK-ERK pathway in cancer. Clin Cancer Res.

[42] Lee S (2016). Epidermal Growth Factor Receptor Signaling to the Mitogen Activated Protein Kinase Pathway Bypasses Ras in Pancreatic Cancer Cells. Pancreas.

[43] Boyer AP, Collier TS, Vidavsky I, Bose R (2013). Quantitative proteomics with siRNA screening identifies novel mechanisms of trastuzumab resistance in HER2 amplified breast cancers. Mol Cell Proteomics.

[44] Luan Z, He Y, Alattar M, Chen Z, He F (2014). Targeting the prohibitin scaffold-CRAF kinase interaction in RAS-ERK-driven pancreatic ductal adenocarcinoma. Mol Cancer.

[45] Golan T (2015). RNAi therapy targeting KRAS in combination with chemotherapy for locally advanced pancreatic cancer patients. Oncotarget.

[46] Parvani JG, Jackson MW (2017). Silencing the roadblocks to effective triple-negative breast cancer treatments by siRNA nanoparticles. Endocr Relat Cancer.

[47] Young SW, Stenzel M, Yang JL (2016). Nanoparticle-siRNA: A potential cancer therapy?. Crit Rev Oncol Hematol.

[48] Jackson EL (2001). Analysis of lung tumor initiation and progression using conditional expression of oncogenic K-ras. Genes Dev.

[49] Olive KP (2004). Mutant p53 gain of function in two mouse models of Li-Fraumeni syndrome. Cell.

[50] Hingorani SR (2003). Preinvasive and invasive ductal pancreatic cancer and its early detection in the mouse. Cancer Cell.

[51] Smigiel JM, Parameswaran N, Jackson MW (2017). Potent EMT and CSC Phenotypes Are Induced By Oncostatin-M in Pancreatic Cancer. Mol Cancer Res.

[52] Garbe JC (2009). Molecular distinctions between stasis and telomere attrition senescence barriers shown by long-term culture of normal human mammary epithelial cells. Cancer Res.

[53] Cipriano R (2013). FAM83B-mediated activation of PI3K/AKT and MAPK signaling cooperates to promote epithelial cell transformation and resistance to targeted therapies. Oncotarget.

[54] Cipriano R (2011). TGF-beta signaling engages an ATM-CHK2-p53-independent RAS-induced senescence and prevents malignant transformation in human mammary epithelial cells. Proc Natl Acad Sci USA.

[55] Junk DJ, Cipriano R, Stampfer M, Jackson MW (2013). Constitutive CCND1/CDK2 activity substitutes for p53 loss, or MYC or oncogenic RAS expression in the transformation of human mammary epithelial cells. PLoS One.

[56] Kan CE, Cipriano R, Jackson MW (2011). c-MYC functions as a molecular switch to alter the response of human mammary epithelial cells to oncostatin M. Cancer Res.

[57] Liu H (2010). Cancer stem cells from human breast tumors are involved in spontaneous metastases in orthotopic mouse models. Proc Natl Acad Sci USA.

[58] Junk DJ (2017). Oncostatin M promotes cancer cell plasticity through cooperative STAT3-SMAD3 signaling. Oncogene.

[59] Reimand J (2016). g:Profiler-a web server for functional interpretation of gene lists (2016 update). Nucleic acids research.

[60] Reimand J, Arak T, Vilo J (2011). g:Profiler–a web server for functional interpretation of gene lists (2011 update). Nucleic acids research.

