# Supplementary material for: A FAM83A Positive Feed-back Loop Drives Survival and Tumorigenicity of Pancreatic Ductal Adenocarcinomas
Source: Sci Rep. 2019 Sep 16;9:13396. doi: 10.1038/s41598-019-49475-5 (PMC6746704; doi:10.1038/s41598-019-49475-5)
Supplement: Supplementary file 1 — Supplementary Information [file 41598_2019_49475_MOESM1_ESM.pdf]

A FAM83A Positive Feed-back Loop Drives Survival and Tumorigenicity of Pancreatic Ductal Adenocarcinomas.

Neetha Parameswaran[1], Courtney A. Bartel[1], Wilnelly Hernandez-Sanchez[2], Kristy L. Miskimen[3], Jacob M. Smigiel[1], Ahmad M. Khalil[4] and Mark W. Jackson*[1,5]

Supplementary Figure S1: FAM83A, not FAM83B, is elevated in pancreatic cancers.

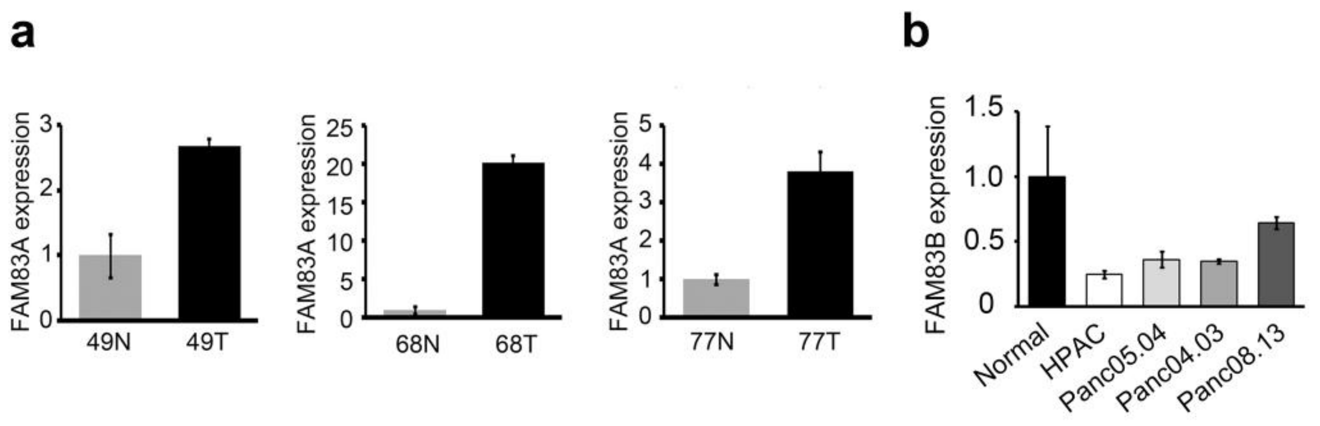

(a) FAM83A expression is elevated in human pancreatic tumor tissue compared to its associated normal pancreatic tissue. (b) FAM83B expression remains unchanged in pancreatic cancer tissue compared to normal pancreas. qRT-PCR for FAM83B in a panel of pancreatic cancer cell lines compared to normal pancreas.

Supplementary Figure S2: Cellular proliferation remains unaltered upon FAM83A knockdown.

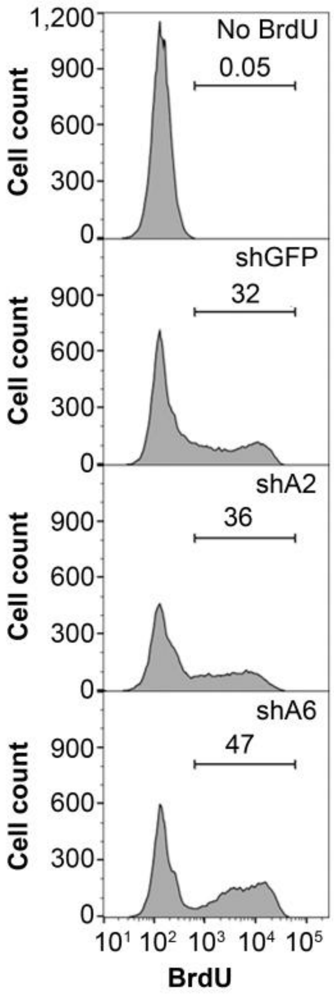

HPAC-shGFP, HPAC-shA2 and HPAC-shA6 cells cultured for 48 h were incubated with nucleoside analog BrdU. Proliferating cells that incorporate BrdU were assessed by flow cytometry as shown in the histogram plots.

a

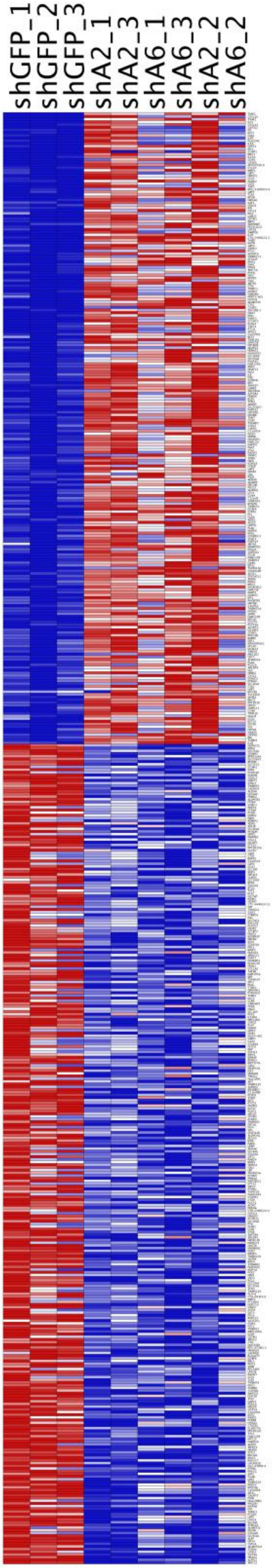

Supplementary Figure S3: RNA sequencing analysis of FAM83A-sufficient and deficient PDAC cells reveals large scale transcriptional changes in gene expression.

b

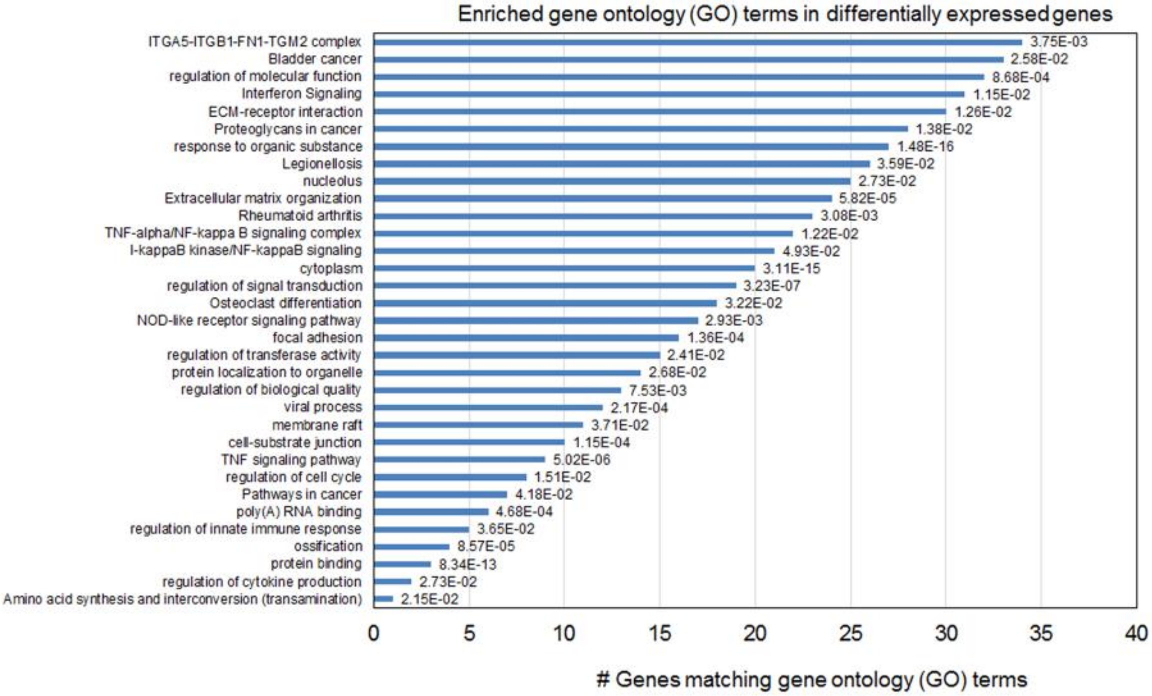

(a) Expression changes (annotated genes only) in the HPAC-shA2 and HPAC-shA6 groups that were in the same direction and with [>1] Log2-fold change relative to shGFP have been presented as a heatmap. (b) Gene set enrichment analysis performed based on the g:Profiler database indicates the pathways that were altered upon FAM83A knockdown.

Supplementary Figure S4: FAM83A expression is regulated independent of KRAS.

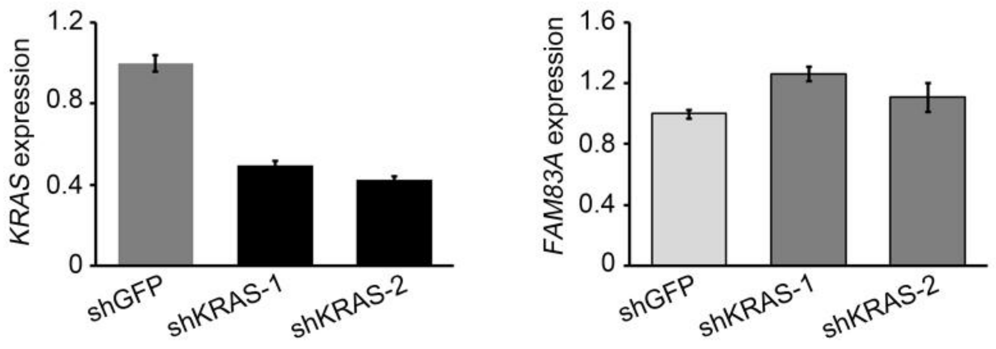

KRAS knockdown using two different shRNAs (shKRAS-1 and shKRAS-2) did not alter FAM83A expression induced by 4 h serum stimulation.

Supplementary Figure S5: Elevated FAM83A expression correlates with an elevated MAPK gene signature.

P-value	Fold Change	Gene
3.67E-10	4.63	NEK2
3.86E-9	2.26	PRKCI
5.36E-9	4.14	PTPRR
5.65E-9	2.20	FLNB
5.00E-8	3.52	HSPA1A
5.22E-8	4.47	IL1R2
1.10E-7	1.97	YWHAZ
1.52E-7	2.00	CACNB3
2.41E-7	2.68	FAM83A
1.09E-6	1.96	RAC1
1.23E-6	3.30	HSPA1B
1.64E-6	3.36	PLA2G10
1.67E-6	1.92	DDIT3
1.89E-6	2.00	PRKCA
4.69E-6	2.56	MAP4K4
5.09E-6	1.74	DUSP6
6.17E-6	1.68	KRAS
6.18E-6	1.75	PAK1
1.72E-5	1.35	IKBKE
1.81E-5	1.94	ZMYND8

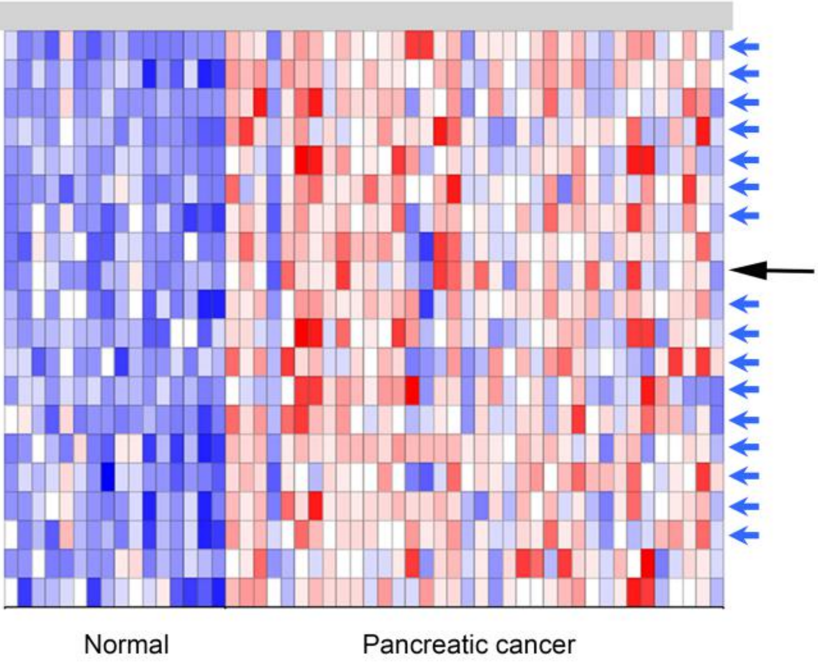

Pei et. al. data(23) on Oncomine was filtered for pancreatic carcinoma vs. normal analysis, followed by the filter for MEK ERK-Kyoto Encyclopedia of Genes and Genomes (KEGG) pathway concept. The figure shows elevated FAM83A expression (black arrow) and a concomitant increase in MEK/ERK signature genes (blue arrows) in pancreatic carcinomas. The fold change and p-value reflect the significant differences between the mean values for each gene in normal pancreas and pancreatic carcinoma.

Supplementary Table S1: Intron-spanning primers used for qRT-PCR were as shown in this table.

***FAM83A* (Human)**	5’ CTCGGACTGGAGATTTGTCC 3’<br>5’ GGAACTCCTCGTCAAACAGC 3’
***FAM83B* (Human)**	5’ ACGTCCAGTGAGCTTCTACG 3’<br>5’ AAGCAATGGACTAGACCTGC 3’
***KRAS* (Human)**	5’ GGGGAGGGCTTTCTTTGTGT 3’<br>5’ GGCATCATCAACACCCTGTCT 3’
***EGR1* (Human)**	5’ GACCGCAGAGTCTTTTCCTGA 3’<br>5’ GTGCCGCTGAGTAAATGGGA 3’
***JUNB* (Human)**	5’ CCTCCCGTTTACACCAACCT 3’<br>5’ GATGCGCTCTTGGTCTTCCA 3’
***FosB* (Human)**	5’ TTCTGACTGTCCCTGCCAAT 3’<br>5’ CGGGGTCAGATGCAAAATAC 3’
***BID* (Human)**	5’ GTCTTTCCAGCACCGCAGA 3’<br>5’ ACCGTTGTTGACCTCACAGTC 3’
***GAPDH* (Human)**	5’ TGCACCACCAACTGCTTAGC 3’<br>5’ GGCATGGACTGTGGTCATGAG 3’
***FAM83A* (Mouse)**	5’ CTGGTCCAGAAGCACACAGA 3’<br>5’ ACATCAGAGATTTGGTCCGC 3’
**GAPDH (Mouse)**	5’ CGTATTGGGCGCCTGGTCAC 3’<br>5’ ATGATGACCCTTTTGGCTCC 3’
